# Enhancing decision quality through computer-based decision aids: how promotional interventions and Need for Cognition shape effectiveness in online consumer choices

**DOI:** 10.3389/fpsyg.2025.1576319

**Published:** 2025-10-29

**Authors:** Claudia Vogrincic-Haselbacher, Isabelle Behlau, Joachim I. Krueger, Katja Corcoran, Brigitta Lurger, Ursula Athenstaedt

**Affiliations:** ^1^Department of Psychology, University Graz, Styria, Austria; ^2^Department of Psychology, Bundeswehr University Munich, Bavaria, Germany; ^3^Department of Cognitive and Psychological Sciences, Brown University, Providence, RI, United States; ^4^Institute of Civil Law, Foreign Private Law and Private International Law, University Graz, Styria, Austria

**Keywords:** Need for Cognition (NFC), computer-based decision aid (CDA), consumer behavior, information processing, decision quality

## Abstract

**Introduction:**

Consumers increasingly face overwhelming amounts of information when making online contractual decisions, particularly as e-commerce continues to expand the scope and complexity of available information. While this digital transformation promises greater transparency, many consumers struggle to process complex information effectively, leading to suboptimal decision making.

**Methods:**

In a simulated online contracting scenario (N = 206), we investigated whether providing and promoting computer-based decision aids (CDA) could enhance decision quality. Additionally, we examined how individual differences in Need for Cognition (NFC), a trait characterizing one's tendency to engage in and enjoy effortful cognitive activities, moderates CDA effectiveness.

**Results:**

We find that promotional nudges increase the adoption of CDA and that they subsequently improve decision quality. These benefits are primarily seen among participants with low NFC, suggesting that decision aids are most valuable for individuals less inclined toward systematic information processing.

**Discussion:**

These findings have significant implications for personalizing digital decision support systems and advancing consumer protection in increasingly complex online marketplaces.

## 1 Introduction

When making online purchase decisions, consumers face an overwhelming amount of information from multiple sources, which can lead to a state of information overload (Eppler and Mengis, 2008; Lurie, 2004). Consumers' affective responses may suffer as well, as they feel less confident and less satisfied with their decisions (Lee and Lee, 2004). This compromised subjective state is often referred to as consumer confusion (Kasper et al., 2010). Consumers' inundation with data does not only lead to confusion but can also significantly impair decision making processes, even to the point of decision paralysis (Hwang and Lin, 1999). The growth of online shopping has accelerated globally, a development further fueled by the COVID-19 pandemic (Ma et al., 2020). As a result, vulnerable groups of consumers, such as older adults or individuals with low media literacy, have entered the online market, which makes finding solutions to decision making problems a task of great urgency (Rydzewska et al., 2024; Strough and Bruine de Bruin, 2020).

Decision-aiding techniques are valuable tools to assist individuals in navigating the available information and making informed choices. Decision aids leverage a range of technologies, from simple devices that aid routine decision situations to complex intelligent systems that perceive and respond to context (Adya and Phillips-Wren, 2020). As consumers' purchasing decisions typically require comparisons among multiple products and attributes (Rydzewska et al., 2024), computer-based decision aids (CDA) offer a promising strategy for reducing the stress associated with choosing between potentially conflicting alternatives and for improving decision quality (Adya and Phillips-Wren, 2020; Phillips-Wren and Adya, 2020; Häubl and Trifts, 2000; Sheehan and Sherman, 2012). CDA include comparison matrices that organize product attributes in standardized formats, which have been shown to have strong favorable effects on both the quality and the efficiency of purchasing decisions in multi-attribute choice tasks (Häubl and Trifts, 2000). Recommendation systems that filter options based on user preferences have also demonstrated significant effects on customers‘ intention to adopt by increasing cognitive and emotional trust (Komiak and Benbasat, 2006). More sophisticated CDA that involve automated decision making by artificial intelligence show promise in financial and healthcare contexts (Zhang et al., 2020), though individuals have mixed opinions about fairness and usefulness of automated decision making (Araujo et al., 2020).

Although the potential benefits of computer-based decision aids are well-established, a significant challenge remains in translating these theoretical advantages into real-world effectiveness. Two critical gaps in the existing literature limit our understanding of how to maximize CDA impact in consumer contexts. First, despite evidence that decision aids can improve decision outcomes, many consumers either fail to use available aids or use them inappropriately which represents a substantial barrier to realizing the full potential of technological decision support. Second, CDA literature has largely overlooked individual psychological characteristics in determining CDA effectiveness. Most research assumes uniform benefits across all users, failing to account for differences in information processing preferences (Motorny et al., 2022; Rydzewska et al., 2024). By investigating how Need for Cognition moderates the relationship between CDA use and decision quality, our research addresses this theoretical gap, while providing insights for more personalized decision support systems. We outline the proposed contribution in the following paragraphs.

Research has demonstrated the advantages of CDA for decision making (Adya and Phillips-Wren, 2020; Phillips-Wren et al., 2009). However, there is evidence of misuse of such aids by decision makers. Whereas the term “use” pertains to the voluntary utilization of a decision aid to support a task, the term “misuse” indicates either an excessive dependence on the aid, a failure to utilize the aid adequately, or a disregard of the recommendations generated by the aid (Adya and Phillips-Wren, 2020). For instance, in a preliminary study (see Supplementary materials for details), we found that a considerable portion of participants (18%) was reluctant to use the decision aids (standardized-information-sheets, CDA) provided to them, although these aids might have been helpful in enhancing decision quality (see supplementary information for details). Misuse of decision aids by discounting advice from decision aids and/or giving excessive weight to the own judgment, has been shown to lead to biased, misguided and overall suboptimal decisions (Adya and Phillips-Wren, 2020; Krueger, 2003). Misuse of decision aids has been linked to various factors, such as mismatches between task requirements and the capabilities of the technology, as well as specific properties of the technology itself (for an overview see Adya and Phillips-Wren, 2020; Phillips-Wren and Adya, 2020).

Research has examined user acceptance of CDA by employing the Technology Acceptance Model (TAM; Davis, 1989; for an overview see Tao et al., 2020), which is a variant of the Theory of Planned Behavior (Ajzen, 1985). The TAM looks to explicit behavioral intentions as the most proximal antecedent of technology use. An individual's general attitude is a more distal antecedent, which in turn depends on the perceived ease of use and perceived usefulness of the technology. The most recent versions of the TAM further incorporate subjective norms as predictors, in order to capture the impact of the perceived beliefs of important others regarding the use of technology (TAM2; Venkatesh et al., 2003). Finally, the model recognizes the role of facilitating conditions in order to capture an individual's perception of the availability of the resources necessary for using the technology (Universal Theory of Acceptance and Use of Technology, UTAUT; Venkatesh et al., 2003). Meta-analytic and longitudinal evidence shows that perceived usefulness, perceived ease of use, and subjective norms robustly drive intention and actual use (King and He, 2006; Schepers and Wetzels, 2007; Venkatesh and Davis, 2000). Against this theoretical background, we expect that promotional messages highlighting key acceptance factors will increase CDA adoption. Specifically, our promotional intervention targets three core TAM constructs: perceived usefulness (by emphasizing how the CDA improves decision outcomes), perceived ease of use (by highlighting the simplicity of the tool), and subjective norms (by referencing consumer protection authority recommendations). Addressing these factors should enhance positive attitudes toward the CDA, thereby increasing behavioral intentions and actual use. *We hypothesize that when individuals are explicitly encouraged to use a CDA, they are more likely to do so (H1)*.

Beyond these observations, Cognitive Load Theory (CLT; Sweller, 1988; Sweller et al., 1998) provides a process-level account of why CDAs should help consumers in complex online choices. Complex choice environments such as a multi-attribute contracts impose high intrinsic load (the irreducible complexity of attributes, pricing contingencies, and trade-offs) and often considerable extraneous load (format inconsistencies, scattered information, and search frictions) on limited-capacity working memory: as a consequence, performance deteriorates as total load approaches or exceeds capacity (Sweller et al., 1998). CDA target extraneous load by structuring attributes into comparable formats (e.g., standardized comparison matrices), sorting and filtering options to identify superior candidates, pruning irrelevant alternatives, and standardizing cost computations and units, thereby freeing resources for core evaluation and more accurate trade-offs (Diehl et al., 2003; Lee and Cho, 2005; Murray and Häubl, 2008; Phillips-Wren and Adya, 2020). This mechanism dovetails with the effort–accuracy framework (Payne et al., 1997), which predicts that decision makers adapt their strategies to balance cognitive effort against expected accuracy. By lowering the marginal effort per unit of accuracy, CDAs should either shift users toward more compensatory, accuracy-enhancing strategies or deliver higher accuracy at the same effort. Accordingly, *we predict that access to a CDA will improve decision quality (H2a) and reduce subjective confusion (H2b) to the extent that the aid meaningfully reduces extraneous load and coordination costs under high-information conditions*.

While existing research has primarily focused on the design and general effectiveness of decision aids, a critical gap remains in understanding how individual cognitive characteristics moderate CDA effectiveness. Few studies have empirically examined the role of individual traits in the acceptance and the effectiveness of CDA (Adya and Phillips-Wren, 2020). Most research settles for a “one-fits-all” approach, assuming that CDA will benefit users on average (Johnson et al., 2012). This assumption ignores fundamental differences in how individuals process information and make decisions. Yet, exploring such individual characteristics is important for practical and theoretical reasons. From a practical standpoint, certain individual characteristics might limit the effectiveness of CDA, or they could even produce adverse effects in some subgroups, leaving consumers with unintended outcomes (Ingendahl et al., 2021). Furthermore, research might inform providers or administrators on how to tailor CDA to individual needs, resulting in a stronger impact on consumer behavior (Ben-Shahar and Porat, 2019; Lurger, 2021; Vogrincic-Haselbacher et al., 2021). From a theoretical perspective, a potential moderation by a given personality trait would shed further light on the cognitive processes associated with the acceptance and effectiveness of CDA. Our study addresses this gap by investigating how the Need for Cognition (NFC; Cacioppo et al., 1983) moderates the relationship between CDA use and decision quality.

Among various individual difference variables that could potentially moderate CDA effectiveness, we consider NFC as being of the greatest theoretical and practical interest because it directly relates to information processing preferences (Cacioppo et al., 1996). NFC is conceived of as trait-like willingness to engage in cognitively demanding tasks, thus reflecting dispositional differences in intrinsic cognitive motivation (Fleischhauer et al., 2010). Being an instance of a dual-process theory of cognition (Evans, 2008), the NFC model assumes that only individuals high in NFC enjoy the experience of thoughtful reflection even if it requires time and effort (e.g., Butler et al., 2003; Sicilia and Ruiz, 2010; Levin et al., 2000). By contrast, individuals with a low NFC tend to settle for superficial processing, relying on easily perceivable cues and judgmental heuristics when they are accessible (Cacioppo et al., 1983, 1996). It has been shown that high NFC can reduce the influence of superficial processing, such as overly simplistic heuristics, on decision making (Cacioppo et al., 1996). Unlike broader personality dimensions (e.g., Big Five traits) that may have indirect or unclear relationships to CDA use and effectiveness, NFC specifically captures individual differences in cognitive motivation which is fundamental to how individuals interact with decision aids. In fact, CDA itself can be viewed as decision heuristic, one that offers a psychologically undemanding alternative to the otherwise necessary deliberate exploration and evaluation of choice options (Carnevale et al., 2011; Smith and Levin, 1996; Verplanken, 1993; Weinmann et al., 2016). A CDA heuristic would presumably work best for individuals low in NFC, saving them the disagreeable cognitive work of thoughtfully searching and processing the necessary information (Dinner et al., 2010; Haugtvedt and Petty, 1992; Ingendahl et al., 2021).

Critically, building on the established relationship between NFC and information processing strategies (Cacioppo et al., 1983), we assume that CDA serve as cognitive shortcuts that are differentially valuable depending on individuals' intrinsic motivation to engage in effortful thinking. *More specifically, we hypothesize that individuals low in NFC limit their engagement with the available information and thus benefit from the simplified decision structure provided by the CDA. By contrast, individuals high in NFC prefer to engage with the available information, and thus make a good decision regardless of the availability of CDA (H3)*.

To test these predictions, we asked participants to review an online contract decision scenario in which they had to choose a cellular service contract from a set of available options. The cellular service market offers an excellent testing ground for our research. Economically, this market is large and it carries a high information and choice load (Bar-Gill and Stone, 2009; Kasper et al., 2010; Turnbull et al., 2000). Consumers need to navigate a highly complex information environment characterized by a multi-layered pricing structure, diverse service options, and high economic stakes, which provides the necessary conditions to test the effectiveness of CDA.

## 2 Materials and methods

### 2.1 Sample

We recruited 210 German-speaking participants from various sources (e.g., social networks, university mailing lists, and word-of-mouth recommendation). Considering the overall task completion time in seconds (s), four participants fell more than three standard deviations above the mean (*M* = 692.1, *SD* = 406.3). The data of these participants were considered extreme outliers and excluded from further analysis. The final sample consisted of 206 participants (129 females, 77 males) ranging in age from 18 to 63 years (*M* = 26.9, *SD* = 8.4, *Mdn* = 25). According to a sensitivity analysis with G^*^Power (Faul et al., 2007), this sample is large enough to detect effect sizes of β = 0.192 or greater with a power of 0.80 (Sensitivity analysis settings: *F* test, Linear multiple regression: fixed model, R^2^ deviation from zero, α = 0.05, power = 0.80, *N* = 206, tested predictors = 1, resulting in *f*^2^ = 0.038 or β = 0.192). Most participants (93%) held an Austrian university entrance diploma. The most frequent household income level (55%) was “up to 1,000 euros” net per month, followed by 25% with a household income level of 1,000 to 2,000 euros, and 20% with a household income of 2,000 and above. After completing the study, participants (optionally) received a raffle ticket for one of ten € 50 gift vouchers from a popular online shop.

### 2.2 Decision-making task

We created a three-part survey in Limesurvey GmbH. In the first part, participants gave their informed consent and completed a questionnaire on demographic data. In the second part, participants were forwarded to an external page featuring the main task of choosing a particular cellular service contract among a set of different options. In doing so, we employed a decision task we had developed for and successfully used in previous work (Vogrincic-Haselbacher et al., 2021). In this task, participants are asked to proceed with their decision as they would if they were actually choosing a cellular service contract. Participants are also asked to base their decision on a particular pattern of consumption (user profile) for a period of 2 years. We used a user profile instead of participants' actual consumption behavior to minimize the influence of previous experience and to prevent participants from drawing conclusions not based on the given information. The user profile we presented contained information about an average monthly consumption of voice call minutes, text messages, and internet data transfer (360 min, 45 text messages and 3,100 MB data).

**Figure 1:**
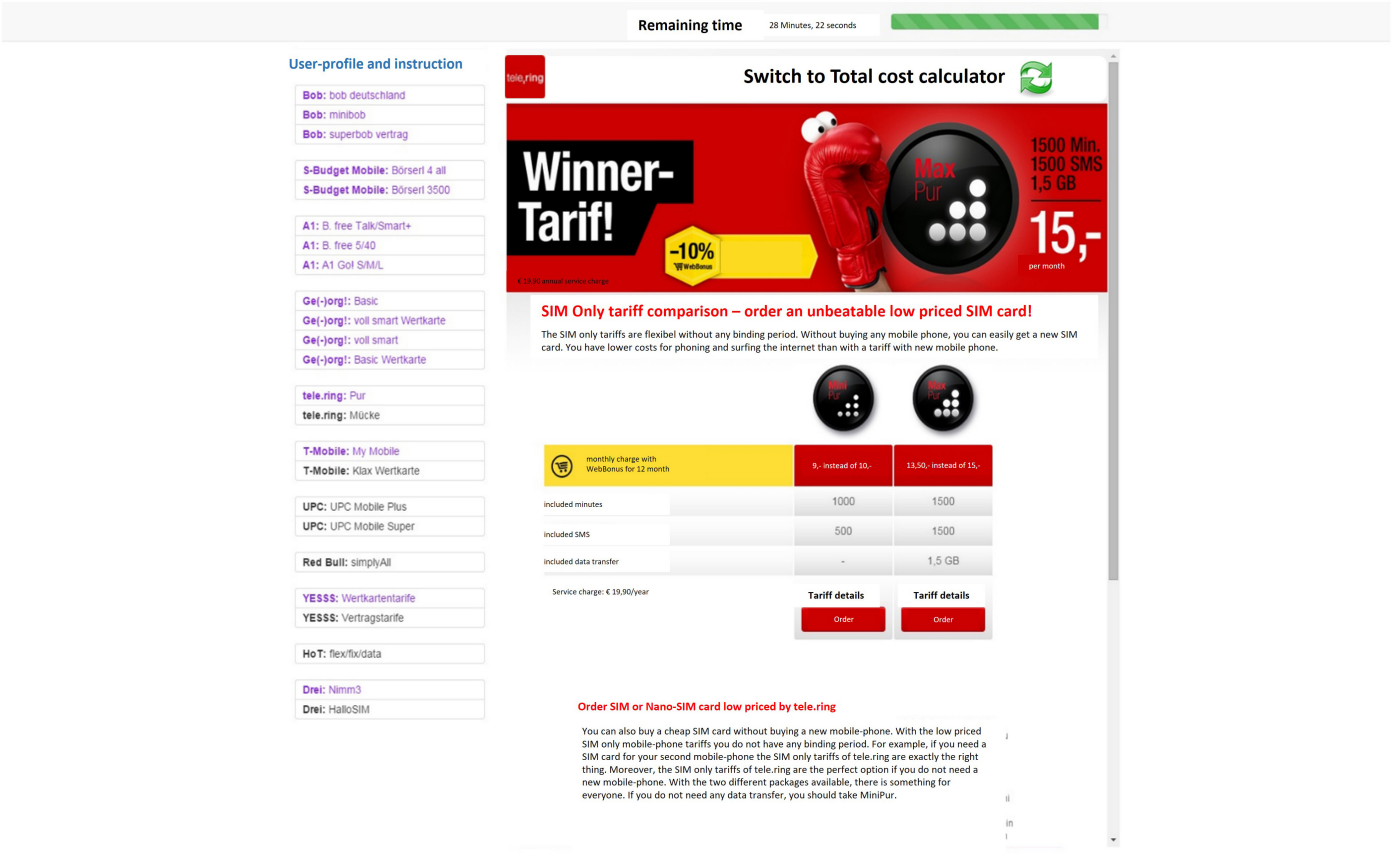
Example view of the web-platform featuring the decision task. The bar on the left side of the screen provides links to all contract options available. By clicking on any of the links, the particular offer got displayed at the center of the screen.

The task featured a web-platform with hyperlinks to different cellular service contract options to choose from (see Figure 1). These hyperlinks were presented in random order in a side bar on the left side of the screen. By clicking on any of these links, participants could browse the information regarding the corresponding offer. Each offer mimicked an actual offer as it appeared on the web in terms of website design and amount of information provided. Specifically, each contract offer included information regarding monthly costs, the number of units allowed per month (minutes, text messages and GB data), the cost per minutes of excess usage, text messaging or GB data, additional costs associated with the activation, service charge and data speed. Some minor changes were made for practical reasons (e.g., links to irrelevant pages, such as ads and bundle offers were removed). In total, 48 different contract options, from 11 cellular service providers, presented on 24 different pages, reflected the prevailing natural conditions regarding the level of choice and information load.

The upper right corner of the screen showed the time remaining for the completion of the task, which was restricted to 30 min. The 30-min time limit was established through previous uses of the task to ensure participants could complete the task without feeling rushed. In previous research (Vogrincic-Haselbacher et al., 2021), most participants completed the decision task in less than 20 min (*M* = 1177.2, *SD* = 589.1) with even the most thorough participants finishing within the 30-min window. The time limit was therefore set above the previously observed completion times to prevent any sense of urgency while still maintaining experimental control. This approach aligns with best practices in experimental design, where time constraints should be sufficiently liberal to avoid confounding task performance with time stress, while preventing participants from becoming distracted or losing focus during extended decision periods. After making their choice, participants were directed to a virtual shopping basket guiding them through a typical web-based contract conclusion procedure, including the possibility of displaying and accepting the general terms and conditions (GTC).

In the third and final part of the survey, participants were asked to complete the post-decisional measures (see measures section for the details). Finally, participants were debriefed and thanked for their contribution to this research. In a separate survey, they had the opportunity to enter their e-mail address if they wanted to enter a raffle for a small prize.

### 2.3 Experimental conditions

Prior to working on the decision task, each participant was randomly assigned to one of two experimental conditions or one control condition using Limesurvey's built-in group randomization script. This random assignment occurred automatically upon entering the study platform via the central study link, ensuring true randomization that was independent of recruitment source or any participant characteristics.

**Figure 2:**
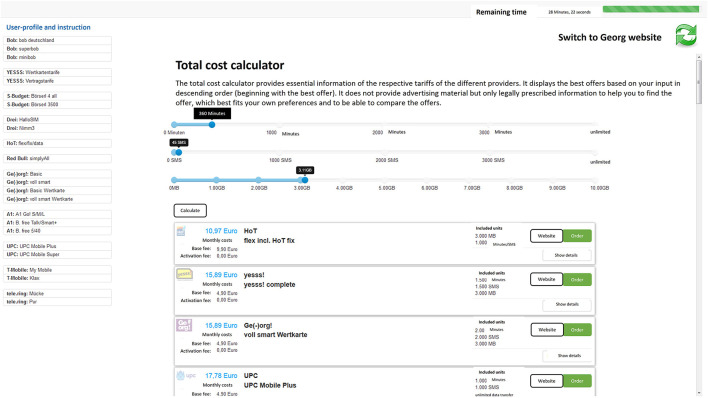
Example view of CDA. The bar on the left side of the screen provides links to all contract options available. Participants could either click on any of the links to view a particular offer or switch to the CDA. After providing basic information on the desired voice minutes, text and data the contract should include (i.e., user profile), participants got a list with all contracts available, ranked from the optimal to the worst offer.

Participants in the control group worked on the task as described above without any decision aids being provided (*noAid*). In one experimental condition (*CDAadd)*, participants were provided with a CDA alongside the information on the web-platform as described above. This CDA comprised a total cost calculator (TCC) designed to simplify and facilitate the choice process. Participants had the option to switch between websites and the TCC at any time with the press of a button at the top right of the screen. The design and the algorithms employed for the TCC followed directly from our definitions of essential contract features and an optimal contract decision, which means that the results it produced reflected these parameters (see section decision quality for more details). By clicking on the TCC button, an input mask required participants to specify the desired minimum of call minutes, text messages, and data the contract should include. After clicking the “calculate” button, participants were presented with a list of available contracts, ordered by monthly costs, starting with the optimal option available (see Figure 2). Monthly costs and the units included per month (minutes, text messages, and GB data) were presented for each available option. By clicking on a “details” button, participants could request further information for each contract, such as exceeding costs per min, text message or GB data, activation fee, service charge, and data speed.

In a second experimental condition (*CDAprom)*, participants proceeded as in the *CDAadd* condition. The critical addition was a pop-up window, which appeared after participants clicked on the first contract offer. Within that pop-up window a promotional message was displayed to encourage the use of the TCC. The message was designed based on the assumptions of the TAM model and its extensions (Tao et al., 2020; Venkatesh and Davis, 2000; Venkatesh et al., 2003). According to this model, perceived usefulness, perceived ease of use, and perceived norms are key variables underlying the acceptance of technical devices such as TCC. Consequently, we highlighted the ease of use, the perceived usefulness and the trustworthiness and accuracy of the TCC. We told participants that consumer protection authorities recommend the TCC for such tasks. To ensure that they had read and understood the information given in the pop-up, participants were able to leave the page only after a delay of at least 10 s. Afterwards, participants were presented with the home screen as described above including a switch button to the TCC on the top right of the screen.

### 2.4 Measures

#### 2.4.1 CDA use

To measure whether participants had used the CDA provided in the two experimental conditions, we tracked their mouse clicks during the entire duration of the decision-making task. The dependent variable of CDA use was coded such that it indicated whether the CDA was used when participants had put in the desired amount of voice minutes, text and data, and had hit the calculate button (1 = used; 0 = not used).

#### 2.4.2 Decision quality

To measure decision quality, we calculated the average monthly cost for each offer, considering a range of costs such as the monthly fee, the activation fee, coverage costs, service fees or discounts over a 2-year period based on a specific usage pattern (user profile). The offers were designed so that one of them was optimal in that it had the lowest monthly cost for the given user profile. As the distribution of absolute additional costs had a strong positive skew, we transformed the absolute values into ranks from 1 to 20, with 20 reflecting the lowest additional costs (i.e., being the best possible solution). Decision quality was calculated by relating the decision actually made (the “subject solution”) to the optimal solution (i.e., [1– (optimal solution – subject solution)/(optimal solution); Speier, 2006]). Values ranged from 0 to 1, with 1 representing an optimal decision and values closer to 0 indicating lower quality decisions.

#### 2.4.3 Consumer confusion

We measured the degree to which participants experienced consumer confusion with nine items adapted from the consumer confusion scale (Kasper et al., 2010). Ratings were made on a 6-point scale (1 = *applies not at all* and 6 = *applies completely)* and averaged into a single score (α = 0.89) with higher values representing greater confusion (e.g., “The more I learn about cellular service contracts and providers, the more difficult becomes my choice”).

#### 2.4.4 Need for Cognition

We measured the Need for Cognition with the German version of the 33-item “Need for Cognition” scale (Bless et al., 1994, e.g., “I enjoy thinking about an issue even when the results of my thought will have no effect on the outcome of the issue”). Ratings fell on a 6-point scale (1 = *applies not at all* and 6 = *applies completely)* and were averaged into a composite score (α = 0.89) with higher values indicating a higher Need for Cognition.

## 3 Results

Statistical analysis and visualizations were conducted using R 4.5.0 (R Core Team, 2025) and several associated packages. Specifically, the following packages were utilized: dplyr (Wickham et al., 2023) ggplot2 (Wickham, 2016), car (Fox and Weisberg, 2019), tidyr (Wickham et al., 2025), knitr (Xie, 2025), ggpubr (Kassambara, 2025), emmeans (Lenth, 2025), and tibble (Müller and Wickham, 2025).

### 3.1 Use of CDA

To test whether the promotion of CDA was successful in that it significantly increased CDA use (H1), we ran a binomial logistic regression analysis on the dependent variable CDA use (0 = not used; 1 = used). As predictors we included the dummy coded experimental condition, with the CDA*add* condition serving as the default condition (0 = default; 1 = *CDAprom*). Although we did not specifically hypothesize that NFC would influence the usage rate, we chose to include NFC (mean centered) as an exploratory variable in the analysis.

The binomial logistic regression model was statistically significant, χ^2^_(2)_ = 14.76, *p* < 0.001, Nagelkerke's *R*^2^ = 0.891, with an overall percentage of accuracy in classification of 84.6%. Among the predictors entered into the model, experimental condition predicted CDA use, *b* = 1.89, *SE* = 0.59, *p* = 0.001, while NFC showed no significant main effect, *b* = 0.57, *SE* = 0.44, *p* = 0.197. In the *CDAprom* condition, the likelihood of using the CDA was increased as compared to the default condition (*CDAadd*), *OR* = 6.61, 95% *CI* (2.1, 20.98). The predicted probabilities for using CDA were 0.73 (95% CI [0.60,0.82]) for *CDAadd* and 0.95 (95% CI [0.86,0.98]) for *CDAprom*.

### 3.2 Decision quality, experimental condition, and need for cognition

To test whether experimental condition predicted decision quality (H2a) and whether NFC moderated potential differences in decision quality (H3), we ran a multiple regression analysis on decision quality. For the effect of experimental condition, we included two dummy coded variables (*CDAadd, CDAprom*), with the no-aid condition serving as the default category. The first dummy variable coded the effect of providing a CDA (*noAid* = 0, *CDAadd* = 1, *CDAprom* = 0), the second dummy variable coded the effect of promoting the CDA (*noAid* = 0, CDA*add* = 0, *CDAprom* = 1). Further, centered Need for Cognition scores (NFC; *M* = 0.00, *SD* = 0.59, range = [−1.94, 1.24]) and the two corresponding interaction effects (NFC^*^CDAadd, NFC^*^CDAprom) were included as predictors. Due to our coding scheme, the slope of NFC reflects the effect in the default condition. The interaction term signals any changes of this slope in the *CDAadd* or the *CDAprom* condition.

For reference, in our sample, the non-standardized NFC score showed a mean of 4.18 (*SD* = 0.59; *min* = 2.24, *max* = 5.42) and was approximately normally distributed (K-S-Test = 0.046, *p* = 0.200). Although formal population norms for NFC are not established, in their seminal paper, Cacioppo and Petty (1982) found that NFC scores tend to follow a normal distribution in the general population, with individual differences influenced by factors such as education, personality traits, and cognitive styles. More recent studies drawing on community or population-representative samples (e.g., Grass et al., 2023) suggest central tendencies near the scale midpoint with ample variability, supporting the view that our observed distribution is typical for adult samples.

**Figure 3:**
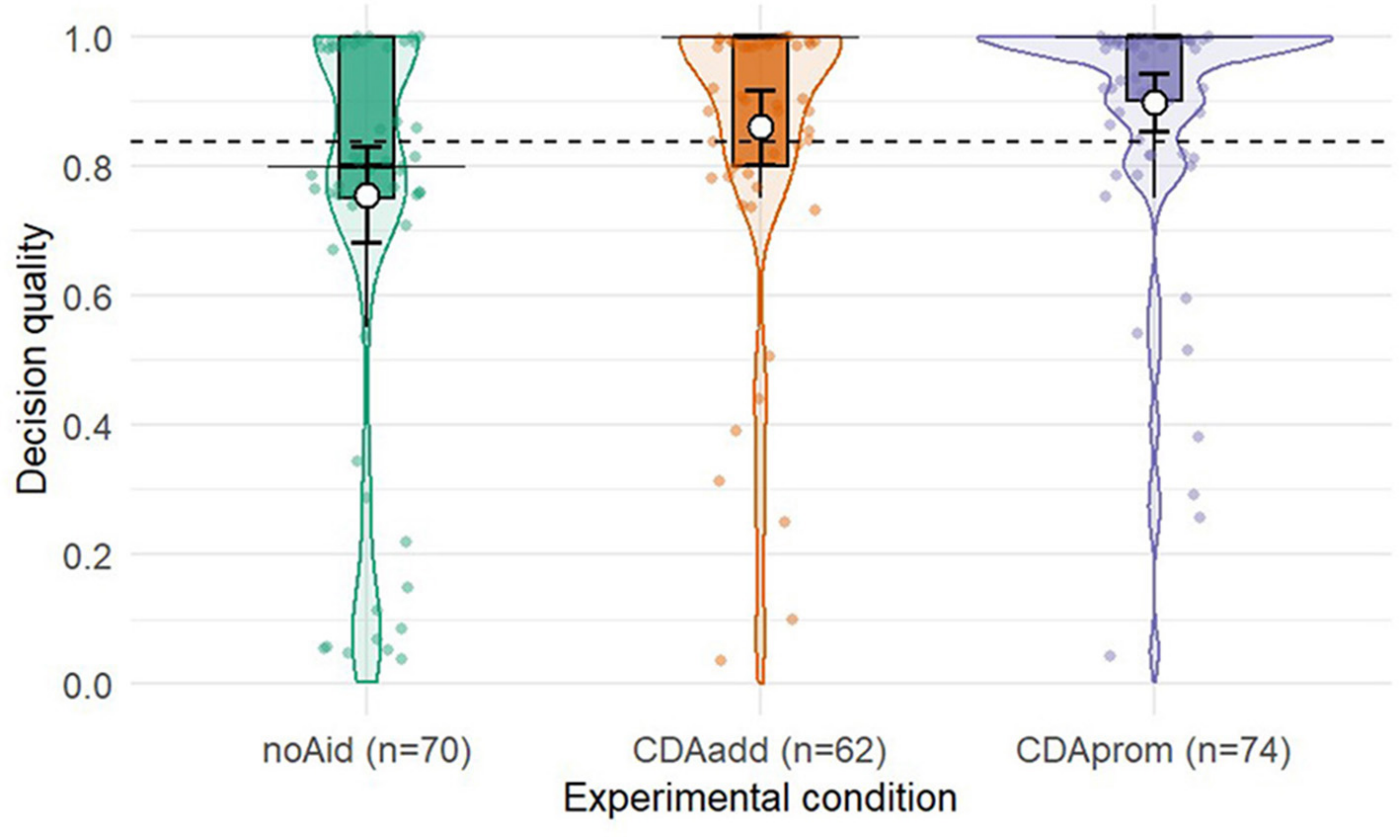
Distribution of decision quality across experimental conditions (noAid, CDAadd, CDAprom). Violin plots show the full distribution of individual scores with superimposed boxplots and slightly jittered raw data points. Black horizontal bars indicate the median, black points the mean, and vertical black lines represent 95% confidence intervals around the mean. The dashed horizontal line denotes the overall mean across all conditions. Sample sizes for each condition are shown on the x-axis.

The overall regression model was significant, *F*_(5, 200)_ = 4.97, *p* < 0.001, *R*^2*corr*.^ = 0.088. The model revealed significant main effects of both decision aid conditions at mean levels of Need for Cognition (NFC). Compared to the *noAid* group, participants in the *CDAadd* condition showed higher decision quality [β = 0.11, *SE* = 0.04, *t*_(200)_ = 2.53, *p* = 0.012], as did participants in the *CDAprom* condition [β = 0.15, *SE* = 0.04, *t*_(200)_ = 3.57, *p* < 0.001]. NFC itself positively predicted decision quality in the *noAid* group [β = 0.15, *SE* = 0.05, *t*_(200)_ = 3.19, *p* = 0.002]. Parameter estimates can be retrieved from Table 1; Figure 3 depicts the distribution of decision quality as a function of experimental condition.

**Table 1:** Multiple regression analysis on decision quality.

**Effect**	**β**	***SE***	***t***	***p***	**95%** ***CI***	
					***LL***	***UL***
(Constant)	0.753	0.029	25.83	< 0.001	0.695	0.810
CDAadd	0.108	0.043	2.53	0.012	0.024	0.192
CDAprom	0.145	0.041	3.57	< 0.001	0.065	0.225
NFC	0.152	0.048	3.19	0.002	0.058	0.247
NFCxCDAadd	−0.076	0.075	−1.01	0.312	−0.223	0.073
NFCxCDAprom	−0.154	0.067	−2.32	0.022	−0.285	−0.023

*N* = 206. NFC, Need for Cognition (centered); β, standardized regression coefficient; SE, standard error; CI, confidence interval; LL, lower limit; UL, *upper limit*.

The interaction between NFC and *CDAadd* was not significant [β = −0.08, *SE* = 0.07, *t*(_200)_ = −1.01, *p* = 0.310], indicating that the slope of NFC did not differ between *CDAadd* and *noAid*. By contrast, the interaction between NFC and *CDAprom* was significant [β = −0.15, *SE* = 0.07, *t*_(200)_ = −2.32, *p* = 0.022]. This pattern suggests that while NFC strongly predicted decision quality in the *noAid* condition, its effect was attenuated when the decision aid was promoted.

To unpack the interaction between NFC and decision aiding, we conducted simple-slopes analyses in two complementary steps. First, we estimated the slope of the association between NFC and decision quality within each condition (*noAid, CDAadd, CDAprom*). Second, we tested conditional pairwise differences between conditions at representative NFC levels (−1 SD, mean, +1 SD), quantifying how the effect of the CDA varies across the NFC continuum.

Simple-slope tests of the association between NFC and decision quality within each condition, showed a clear attenuation pattern under decision aids. While in the *noAid* condition, higher NFC was associated with higher decision quality (as reported above), in the *CDAadd* condition, the NFC slope was smaller and not statistically significant [slope = 0.08, SE = 0.06, *t*_(200)_ = 1.33, *p* = 0.185, 95% CI (−0.037, 0.190)]. In the *CDAprom* condition, the slope was essentially zero [slope = −0.002, SE = 0.05, *t*_(200)_ = −0.04, *p* = 0.972, 95% CI (−0.093, 0.090)]. These results indicate that without an aid, decision quality increases with NFC, whereas providing and especially promoting the CDA, reduces or eliminates the dependence of decision quality on NFC.

**Figure 4:**
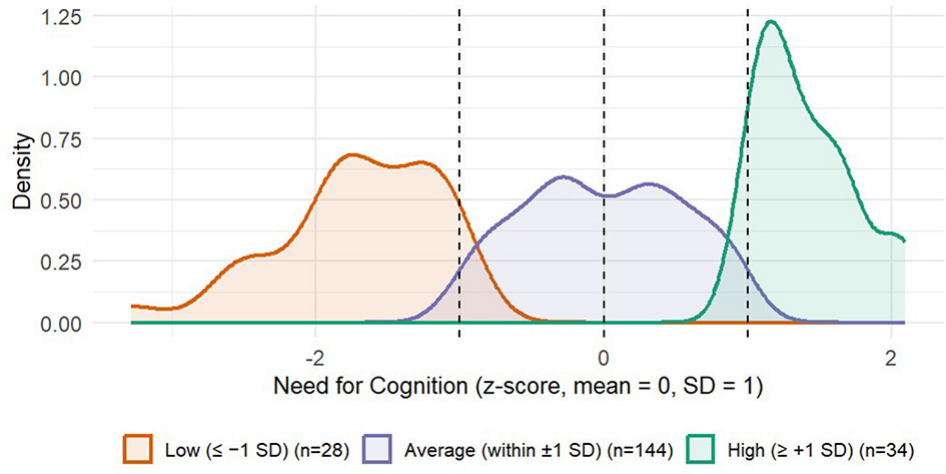
Smoothed kernel density estimates of centered Need for Cognition (scores). Curves are shown for three NFC subgroups defined at ±1 SD (Low, Average, High). Dashed vertical lines mark −1 SD, 0 (average), and +1 SD. Axes display NFC (z) on the x-axis and kernel density on the y-axis. Because kernel density estimation smooths each subgroup's scores with bell-shaped kernels, the curves can visually overlap near the ±1 SD cut points even though the underlying groups share no observations; this overlap reflects smoothing beyond each group's exact range rather than data overlap.

Conditional effects of each CDA condition relative to the *noAid* reference group were tested at “low” (scores below 1 *SD* below the mean), “average” (scores within 1 *SD* of the mean), and “high” (scores above 1 SD above the mean) levels of NFC. Cut-off points fell at −0.591 (low, *n* = 28, 13.6%),0.0003 (average, *n* = 144, 69.9%), and 0.591 (high, *n* = 34, 16.5%). Figure 4 depicts the distribution of the standardized NFC score separated for NFC subgroups. The analysis revealed that individuals low in NFC (Mean −1 *SD*) benefitted from the CDA as indicated by higher decision quality in both *CDAadd* [β = 0.15, *t*_(200)_ = 2.48; *p* = 0.027, 95% CI (0.015,0.209)] and *CDAprom* [β = 0.24, *t*_(200)_ = 4.15; *p* < 0.001, 95% CI (0.109, 163)] compared to the *noAid* condition. As reported above, a similar pattern but with smaller effects arose for individuals with an average level of NFC, also showing higher decision quality in both the *CDAadd* and *CDAprom* condition, compared to the *noAid* condition. As hypothesized, for individuals high in NFC (Mean + 1 *SD*), neither providing [*CDAadd:* β = 0.06, *t*_(200)_ = 1.02; *p* = 0.490], nor promoting a CDA [*CDAprom:* β = 0.05, *t*_(200)_ = 0.96; *p* = 0.532] did affect decision quality, suggesting that these individuals do not need decision aiding in order to do well in the decision task.

**Figure 5:**
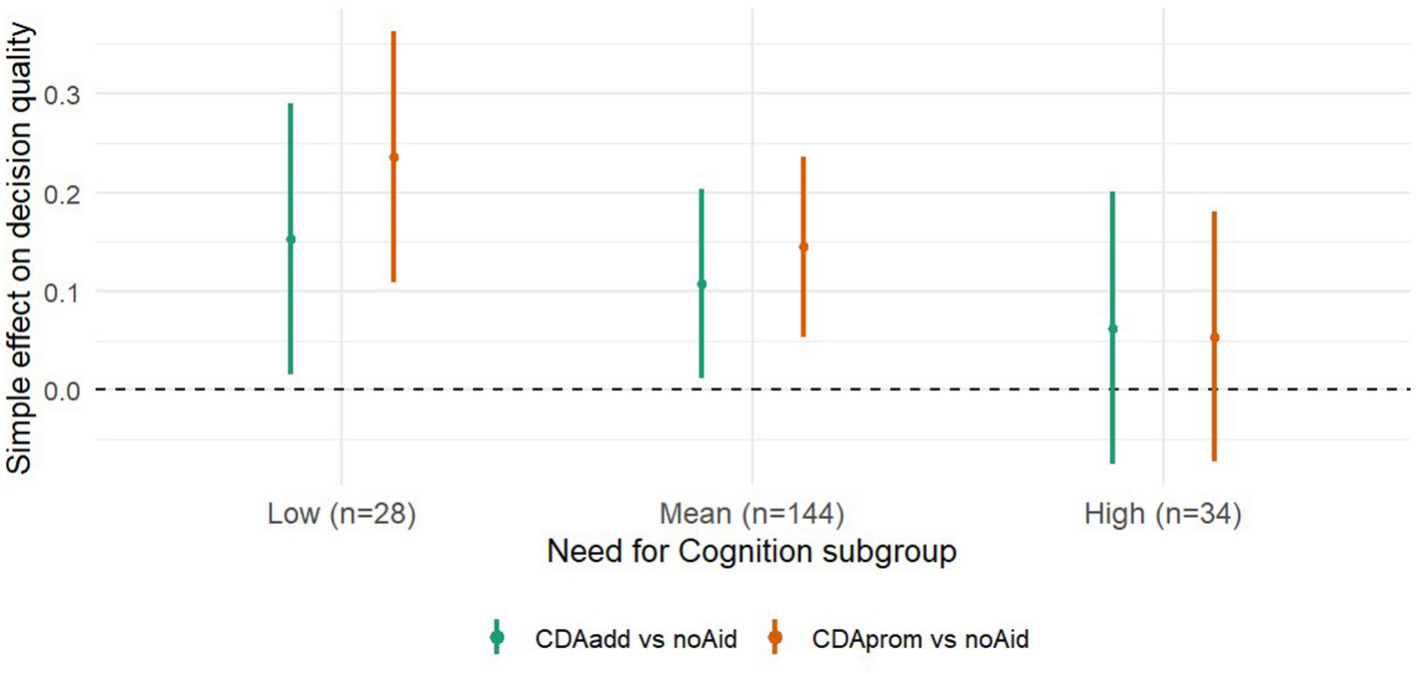
Simple effects of CDA at different levels of Need for Cognition (NFC). The x-axis represents NFC subgroups defined at ±1 SD around the mean. Points represent estimated differences in decision quality between each CDA condition and the noAid reference, with vertical bars showing 95% confidence intervals. The dashed horizontal line denotes zero (no difference). Positive values indicate higher decision quality compared to noAid.

Table 2 provides descriptive statistics while Table 3 provides the results of the pairwise comparisons (simple slopes) of experimental groups at different levels of NFC. Figure 5 illustrates the estimated differences in decision quality at low, average, and high levels of NFC (±1 SD).

**Table 2:** Descriptive statistics for decision quality as a function of experimental condition and Need for Cognition (NFC).

	**Experimental condition**											
	***noAid (n*** = **70)**				***CDAadd*** **(*****n*** = **62)**				***CDAprom*** **(*****n*** = **74)**			
**NFC group**	***n***	**%**	***M***	***SD***	***n***	**%**	***M***	***SD***	***n***	**%**	***M***	***SD***
Low (*n* = 28)	9	4.4	0.48	0.40	6	2.9	0.81	0.22	13	6.3	0.81	0.19
Average (*n* = 144)	49	23.8	0.78	0.29	48	23.3	0.85	0.24	47	22.8	0.89	0.21
High (*n* = 34)	12	5.8	0.85	0.23	8	3.9	0.94	0.11	14	6.8	0.92	0.11

**Table 3:** Pairwise contrasts (simple slopes) of experimental condition at levels of Need for Cognition (NFC).

**NFC subgroup**	***n***	**Contrast**	**β**	***SE***	***t*_(200)_**	***p***	**95%** ***CI***	
							***LL***	***UL***
Low (−1 SD; −0.59)	28	CDAadd	0.153	0.061	2.48	0.027	0.015	0.290
		CDAprom	0.236	0.057	4.15	0.000	0.109	0.363
Average (~ 0)	144	CDAadd	0.108	0.043	2.53	0.023	0.013	0.203
		CDAprom	0.145	0.041	3.57	0.000	0.054	0.236
High (+1 SD; 0.59)	34	CDAadd	0.063	0.061	1.02	0.490	0.074	0.200
		CDAprom	0.054	0.056	0.96	0.532	0.072	0.180

*N* = 206. Contrasts represents the difference between each condition and the reference group noAid. β, standardized regression coefficient; SE, standard error; CI, confidence interval; LL, lower limit; UL, upper limit.

**Figure 6:**
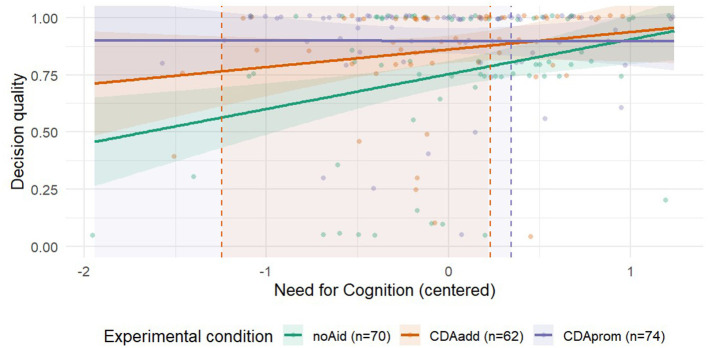
Decision quality as a function of Need for Cognition (NFC) and experimental condition. Solid lines represent fitted regression slopes; shaded bands indicate 95% confidence intervals. Raw observations are lightly jittered to reduce overplotting. Dashed vertical lines mark the Johnson-Neyman (J–N) bounds where the conditional effect of each decision aid condition relative to *noAid* transitions between statistical significance and non-significance. Lightly shaded background regions indicate moderator values for which conditional effects are statistically significant. For *CDAadd*, the J-N bounds lay between NFC = −1.25 and NFC = 0.023. For *CDAprom*, the upper J–N bound lay above the maximum observed moderator value (NFC = 6.49), which implies significance for values below the interior bound (NFC = 0.35), as indicated by the lightly shaded background region.

While the simple slope approach provides interpretable snapshots, it relies on relatively arbitrary moderator values. To ensure robustness of our results, we complemented this analysis with a Johnson-Neyman (J–N) procedure, which identifies the exact regions of the moderator where the conditional effects are statistically significant across the full continuum of NFC. For the comparison between *CDAadd* and *noAid*, the J-N analysis identified a significant region of NFC values between NFC = −1.25 and NFC = 0.23. This suggests that the effect of *CDAadd* relative to *noAid* was contingent on participants' NFC levels, with significant differences observed within this range of NFC. Outside this interval, the difference between *CDAadd* and *noAid* is not statistically significant. For the comparison between *CDAprom* and *noAid*, the analysis identified a lower bound of significance at NFC = 0.35, while the upper bound lies far beyond the observed moderator range (NFC = 6.49). As the upper bound is outside the observed moderator range, the relevant interpretation is a single lower bound indicating that *CDAprom* produces significantly higher decision quality than *noAid* for NFC values below ~ 0.35, with no reliable advantage at higher NFC in the data. In line with the results of the simple slope analysis, the J-N results show that providing and promoting CDA help primarily at lower to average NFC, with promotion extending the region of significant benefit across a broader low-NFC range than provision alone. These results provide further evidence of the moderating role of NFC in the observed effects and are visually represented in Figure 6.

### 3.3 Consumer confusion as a function of experimental condition

We ran a one-way ANOVA to test whether consumer confusion differed across experimental conditions (H2b). There were no significant differences in consumer confusion across experimental conditions, *F*_(2, 203)_ = 0.662, *p* = 0.517. Yet, there was a negative and significant correlation between the experienced confusion and decision quality, *r* = −0.266, *p* < 0.001. Participants who suffered from information load were more likely to make poor choices. Additionally, consumer confusion was negatively correlated with NFC, *r* = −0.331, *p* < 0.001. That is to say, a low NFC was associated with greater consumer confusion.

## 4 Discussion

Despite evidence for the positive impact of computer-based decision aids (CDA) on decision quality, misuse of CDA remains a critical barrier to their effectiveness (Adya and Phillips-Wren, 2020; Phillips-Wren et al., 2009). In an effort to ascertain how the acceptance of CDA can be improved, and using the assumptions of the Technology Acceptance Model (TAM; Davis, 1989; Tao et al., 2020; Venkatesh and Davis, 2000; Venkatesh et al., 2003) as our theoretical framework, we presented a promotional message that highlighted ease of use, perceived usefulness, accuracy and trustworthiness of CDA, to one group of participants while they were working on a simulated online contract conclusion scenario involving multi-attribute decision making. We contrasted the responses of these participants with the responses of participants who were only presented with an unpromoted CDA and participants who had to decide without any CDA and tested whether decision quality differs across groups. We expected that encouraging the use of CDA would increase the proportion of CDA use and decision quality while reducing consumer confusion. Our study demonstrates that the effectiveness of CDA depends critically on both how they are presented to users and individual differences in cognitive motivation. While promotional messaging successfully increased adoption rates across all users, the performance benefits derived from CDA use varied systematically with participants' Need for Cognition (NFC).

### 4.1 Effectiveness of computer-based decision aiding and technology acceptance

The promotional intervention proved highly effective, increasing decision quality significantly in the promotion condition, compared with the unpromoted condition. By highlighting ease of use, perceived usefulness, accuracy, and trustworthiness, promotional messaging enhanced perceived utility and encouraged broader engagement, consistent with the core predictions of the TAM (Davis, 1989; Tao et al., 2020; Venkatesh et al., 2003; Venkatesh and Davis, 2000). Importantly, NFC did not predict initial aid adoption, indicating that promotional messaging increased uptake across the whole cognitive motivation range. This suggests that when individuals are presented with a CDA, their choice to utilize it may be influenced by factors other than their cognitive motivation. These factors could include contextual elements such as promotional messaging, perceived task importance, or social expectations (Bolenz et al., 2022). In a nutshell, providing the aid is necessary yet not sufficient. Promotion grounded in the TAM acts as a catalyst, turning the same tool into broader and more reliable improvements in decision quality.

### 4.2 Individual differences in Need for Cognition (NFC) and the effectiveness of CDA

Our results support a compensatory moderation by NFC. In the absence of an aid, individuals higher in NFC achieved greater decision quality, consistent with their propensity for effortful processing. When the aid was simply provided, this positive association weakened and effectively disappeared when the aid was promoted. This indicates that promotion neutralized the dependence of performance on NFC. Simple-slopes tests and Johnson-Neyman analyses converged on the same pattern, namely that providing and promoting the aid benefited users with low to average NFC, with promotion extending the range of reliable gains further into the lower end of the continuum. At higher Need for Cognition, the aid offered little incremental value because these individuals already performed well without assistance.

This pattern aligns with dual-process theories and established NFC research. Individuals high in NFC prefer systematic information processing (Fleischhauer et al., 2010; Haugtvedt and Petty, 1992; Ingendahl et al., 2021; Cacioppo et al., 1996) and make good decisions regardless of the availability of CDA (Haugtvedt and Petty, 1992; Ingendahl et al., 2021; Fleischhauer et al., 2010). There is evidence that they may even lose interest when decision-making is automated (Sicilia and Ruiz, 2010). Conversely, individuals low in NFC rely on effort-saving heuristics and perceive decision aids as welcome tools that enable good decisions without extensive cognitive investment (Carnevale et al., 2011; Dinner et al., 2010; Smith and Levin, 1996; Verplanken, 1993; Weinmann et al., 2016).

Importantly, while the initial decision to adopt CDA was not related to NFC, once individuals engage with the CDA their underlying cognitive preferences become salient in determining how they process and benefit from the structured information. This means that the divergent interaction patterns are not driven by selective uptake among different NFC groups, but rather by differences in how effectively individuals of varying NFC levels leveraged the aid once they engaged with it. In other words, promotion enhanced both the likelihood of use (independent of NFC) and the performance benefits derived from that use, particularly for lower-NFC individuals. This suggests a stage-specific model where TAM processes drive adoption decisions, while individual difference mechanisms condition effectiveness once the aid is engaged.

Taken together, the results support a “personalize-for-impact” strategy that makes guidance and salience most prominent for users low in NFC while offering optional depth and transparency for those higher in NFC.

### 4.3 The confusion paradox: objective vs. subjective benefits

Although consumer confusion was associated with lower decision quality, we found no significant differences in confusion across experimental conditions. This dissociation between objective decision improvement and unchanged subjective confusion represents a theoretically important finding that challenges simple cognitive-load accounts. While CDA improved decision quality, they did not reduce reported confusion, contradicting our hypothesis based on Cognitive Load Theory (CLT) that decision support should lower extraneous cognitive load (Sweller, 1988; Sweller et al., 1998).

Two complementary mechanisms may explain this pattern. First, individual differences in confusion proneness dominate subjective experience. The substantial negative correlation between NFC and confusion points to a trait-dominated experience of confusion rather than a readily manipulable state. NFC captures relatively stable preferences for engaging in effortful, analytic processing. When individuals who are dispositionally higher in NFC consistently report less confusion across the same decision environment and when confusion remains largely unchanged despite an intervention that demonstrably improves objective accuracy, it seems likely that between-person differences in cognitive motivation drive the subjective feeling of confusion. This aligns with a part of the consumer-confusion literature, which characterizes confusion proneness as a relatively stable predisposition encompassing overload, similarity, and ambiguity sources of confusion (Walsh and Mitchell, 2010). On this view, the aid can restructure information and offload computations, without necessarily shifting their trait-like tendency to feel confused.

Second, metacognitive research shows that subjective ease (fluency) often diverges from objective accuracy (e.g., Alter and Oppenheimer, 2009). CDA may reduce computational demands while at the same time introducing interface and coordination costs. This requires users to monitor aid outputs and integrate recommendations, leaving subjective workload unchanged despite improved outcomes (Perry et al., 2012; Sheehan and Sherman, 2012). Putting these strands together, CDA compensate for decision-making challenges among low-NFC consumers by providing decision structure and systematic support, thereby improving objective performance even when subjective confusion remains. Thus, confusion operates more as a selector of who benefits from CDA than as a mechanism directly altered by the aid in our setting.

### 4.4 Theoretical contributions

We advance understanding of the boundary conditions for CDA effectiveness by demonstrating that individual differences in cognitive motivation fundamentally alter how consumers benefit from technological decision support. Our findings challenge prevailing assumptions about universal CDA benefits by demonstrating that effectiveness is fundamentally contingent on user characteristics. This supports calls for personalized approaches to decision support (Motorny et al., 2022; Rydzewska et al., 2024) and suggests that effective consumer protection requires understanding individual differences in information processing preferences rather than offering generic solutions (Ben-Shahar and Porat, 2019).

While TAM is well-validated as a predictive framework (e.g., King and He, 2006; Schepers and Wetzels, 2007), our study provides novel evidence for its application to decision aid promotion in complex choice environments. Promotional messaging that highlights perceived usefulness, ease of use, and social endorsement increased actual uptake of an otherwise identical CDA (Davis, 1989; Venkatesh and Davis, 2000). Crucially, NFC did not predict aid use, indicating that this acceptance pathway generalized across users. The contribution is a more stage-specific view: TAM processes drive adoption, whereas individual-difference mechanisms (NFC) condition effectiveness once the aid is used. This stage-specific model, where acceptance processes operate independently of effectiveness mechanism, refines our understanding of technology adoption vs utilization in decision support contexts.

Finally, by demonstrating objective benefits without subjective reduction of confusion, our results refine mechanism claims of the CLT in decision aid contexts. CDA appear to bolster decision quality primarily through structural scaffolding rather than by uniformly improving momentary cognitive experience. This theoretical refinement has implications for understanding when and how decision support tools create value.

### 4.5 Practical implications

Our findings demonstrate that the impact of a computer-based decision aid is shaped not only by individual cognitive motivation but also by the strategy through which the aid is introduced (Johnson et al., 2012). As our research shows, simply providing CDA is not enough for decision makers to actually use it (see also Adya and Phillips-Wren, 2020). While the aid itself was identical, promoting it, increased uptake overall and ensured benefits across a wider range of Need for Cognition levels. This aligns with the Technology Acceptance Model emphasizing that communication strategies can shape perceived utility and effectiveness. More broadly, the results highlight that successful implementation of decision aids requires tailoring not only their design but also their introduction to users' motivational profiles.

People must be nudged into using these aids adequately. We have demonstrated that offering a salient reminder on the benefits of such an aid might suffice to raise the proportion of users. Future research could address different nudging techniques that can be successfully integrated into consumer protection legislation. Research should focus particularly on digital nudging techniques, as the increasing use of digital technologies implies that people frequently make important decisions in digital choice environments (Weinmann et al., 2016). Such techniques can include, for instance, providing feedback or anchors, setting defaults, structuring complex screens or providing norms about what other people do or accept (Weinmann et al., 2016; Ingendahl et al., 2021). Even subtle modifications of the choice architecture can influence people's choices and “nudge” them to behave in certain ways. Consequently, a key consideration in such design decisions is that nudges must help people make better choices, which is not always the case in practice.

We found evidence that individual traits, such as the Need for Cognition, impact the effectiveness of CDA, indicating that there are subgroups of consumers (i.e., low in NFC) that require and benefit from decision aiding tools, while others (i.e., high in NFC) won't. This finding has important implications for policy design, suggesting that regulations requiring decision aids may be more effective for some consumers than others, and that targeted approaches may be more efficient than universal mandates (Ben-Shahar and Porat, 2019). Effective consumer decision support requires recognizing that different consumer segments have fundamentally different information processing needs (Ben-Shahar and Porat, 2019; Lurger, 2021). This principle of targeted support aligns with previous research identifying distinct consumer archetypes in complex choice environments. For instance, Vogrincic-Haselbacher et al. (2021) identified three consumer groups with markedly different search behaviors: efficient searchers who focused on optimal options early, hasty deciders who gathered insufficient information, and unfocused searchers who extensively but ineffectively explored options. Notably, both poorly performing groups would likely benefit from decision aids. While hasty deciders need structured guidance to ensure adequate information consideration, unfocused searchers need tools to streamline their search process. This heterogeneity underscores why universal decision aid mandates may be less effective than targeted approaches that match support tools to specific consumer needs and processing styles.

### 4.6 Limitations and direction for future research

Several limitations merit consideration. First, our focus on a single market (cellular services) and decision aid type (total cost calculators) limits the generalizability of the findings. While we chose these contexts for their complexity and practical relevance, future research should examine whether our findings extend to other domains and aid types. Second, although we created a realistic decision environment, the hypothetical nature of choices may limit ecological validity, despite evidence that hypothetical and real decisions often yield similar insights (Ingendahl et al., 2021; Johnson and Bickel, 2002). Lastly, our results are limited to a specific CDA, i.e., total costs calculators (TCC). CDA is a broad category, ranging from simple technologies that aid routine decision situations to highly complex artificial intelligences. We chose a TCC for our study as such calculators are, from a consumer protection perspective, easy to implement and can be beneficial in a wide range of consumer decisions. However, one should be careful in generalizing to other types of CDA.

## 5 Conclusion

As digital marketplaces become increasingly complex, effective decision support becomes more critical for consumer welfare. Our findings demonstrate that while promotional interventions can increase adoption universally, individual differences fundamentally determine who benefits most from these tools. Moving beyond one-size-fits-all solutions toward personalized decision support represents both an opportunity for better consumer outcomes and a necessity for effective consumer protection in the digital age (Ben-Shahar and Porat, 2019). The path forward requires recognizing that consumer decision support is not simply about providing better tools, but about understanding how different consumers interact with those tools and designing both technologies and policies accordingly. Only through such nuanced approaches can we realize the full potential of decision aids to improve consumer welfare in complex choice environments.

## Data Availability

The datasets, analysis scripts as well as supplementary results can be accessed in the Open Science Framework via the link https://osf.io/szctq/.
