# Supplementary material for: Enhancing decision quality through computer-based decision aids: how promotional interventions and Need for Cognition shape effectiveness in online consumer choices
Source: Front Psychol. 2025 Oct 29;16:1576319. doi: 10.3389/fpsyg.2025.1576319 (PMC12605097; doi:10.3389/fpsyg.2025.1576319)
Supplement: Supplementary file 1 [file Supplementary_file_1.docx]

**Supplementary Material**

**Prestudy**

# Introduction

Information overload occurs when decision-makers encounter more information than they can effectively process, leading to impaired decision quality (Jacoby et al., 1974; Bröder and Schiffer, 2006). Research shows that excess information reduces decision accuracy and speed (Jacoby et al., 1974), while also diminishing consumer confidence and satisfaction (Lee and Lee, 2004; Elie‐Dit‐Cosaque, 2015). This state of consumer confusion (Kasper et al., 2010) raises important questions about how to prevent or mitigate the negative consequences of information overload.

To prevent information overload, decision aids can be designed to present information more comprehensibly by highlighting essential product or service attributes. These aids improve information comparability through better content organization and design (Oehler, 2012; Oehler and Reisch, 2008). They come in two forms: passive aids, such as standardized information sheets, and interactive computer-based tools (Breugelmans et al., 2012). In the financial services sector, the European Union has mandated standardized information sheets to help consumers understand complex financial products while simultaneously addressing information asymmetries between businesses and consumers (for an overview see Garcia and van Boom, 2009). These aids achieve their goals by presenting brief, standardized information in a format designed to highlight key product features and facilitate comparison tables (Lee et al., 2012; Stone and Schkade, 1994; Helberger, 2013). Research, although somewhat limited, shows these standardized formats enhance both comprehension and product comparability compared to traditional information sheets (Oehler, 2012).

Computer-based decision aids (CDA) offer another strategy to mitigate information overload (Häubl and Trifts, 2000). These tools go beyond standardizing information by providing interactive features that support cognitively demanding tasks like database searches and criteria-based sorting (Kasper and Lenz, 2005). CDA present essential information concisely, enabling high-quality decisions with minimal effort (Murray and Häubl, 2007), while reducing search costs and better aligning the search process with consumer needs (Diehl et al., 2003; Lee and Cho, 2005). Unlike static standardized information sheets, CDA actively engage consumers in utilizing relevant advice (Breugelmans et al., 2012). For example, the Austrian Telecommunication Act authorizes the creation of an interactive total cost comparison tool to help consumers evaluate service offers, comparing not only fees and pricing mechanisms but also essential contract terms. While government-approved tools are still pending, several private websites already provide cost comparison services for the Austrian market.

The effectiveness of legislative initiatives like providing standardized information and the Austrian Telecommunication Act requires empirical evaluation, but current research remains limited. Most studies focus narrowly on assessing document comprehensibility, format, and usability, rather than examining normatively desirable outcomes like decision quality. A notable exception is Häubl and Trifts’ (2000) early study on computer-based decision aids, which found that an interactive comparison matrix improved both decision quality and efficiency, helping consumers make choices better aligned with their preferences while expending less effort. However, research on how standardized product information sheets affect objective decision quality appears absent from the literature. This lack of comprehensive empirical evidence about how different decision aids impact consumer choices represents a significant gap, particularly given the importance of such findings for consumer protection policy.

The aim of this prestudy was to develop and evaluate decision aids within a high-choice and multi-attribute decision-making context. We studied two types of decision aids – standardized-information-sheets (SIS) and a computer-based decision aid (whole market total cost calculator; TCC) – within an online contract decision scenario that asked participants to select a contract from a set of available mobile-phone service providers. We hypothesized that providing decision aids will improve both objectively quantified decision quality and subjectively experienced information load (consumer confusion).

# Materials and Methods

## Sample

We recruited 216 German-speaking participants from various sources (e.g., social networks, university mailing lists, and word-of-mouth recommendation). To enroll in the study, recruits had to be at least 18 years old, as this is the minimum age to be allowed to sign contracts in Austria. Participants whose overall task completion time fell more than three standard deviations above (or below) the mean (*M* = 708.1, *SD* = 477.6), were considered extreme outliers. The data of 10 participants were identified as extreme outliers based on this rule and excluded from further analysis. The final sample consisted of 206 participants (138 females, 66 males, 2 other) aged 18 to 68 (*M* = 29.5, *SD* = 10.0). According to *a priori* power analysis using G*Power (Faul et al., 2007), a sample of 200 participants was needed to detect medium sized effect with α = .05 and 1 – β = .9.

Most participants held a university-entrance diploma (47.6%), were enrolled at a university (61.7%), had a monthly income of €500 to €1000 (29.1%), and were Austrian nationals (80.1%). After completing the study, participants received a raffle ticket for one of ten €50 Amazon vouchers.

## Decision making task and procedure

The study used a three-part survey designed in Limesurvey. Part one collected informed consent and demographic information from participants. Part two focused on the main experimental task, where participants were redirected to an external platform to select a cellular service contract from multiple options. This decision task, previously validated in our (Vogrincic-Haselbacher et al., 2021), was designed to simulate a real-world contract selection process. To standardize the decision-making context and minimize the influence of prior experience, participants were given a specific user profile to base their decision on. This profile specified monthly usage parameters: 360 minutes of voice calls, 45 text messages, and 3100 MB of data over a two-year period.

The web platform featured a sidebar with randomized hyperlinks to different contract options. Clicking these links allowed participants to view detailed information about each offer, with websites designed to mirror actual market offerings. Each contract page displayed comprehensive information including monthly costs, usage allowances (minutes, texts, and data), overage charges, activation fees, service charges, and data speeds. We made minor modifications for clarity, such as removing irrelevant links to advertisements and bundle offers. The platform presented 48 distinct contract options from 11 service providers, spread across 24 pages, accurately reflecting real-world choice and information conditions. Participants had 30 minutes to complete their selection, with a countdown timer displayed in the upper right corner. After choosing a contract, participants proceeded through a typical online purchase workflow, including the option to review and accept the general terms and conditions.

In part three, participants completed post-decision measurements. The survey concluded with a debriefing session and acknowledgment of their participation. Participants could then opt into a separate survey to provide their email address for a prize drawing.

## Experimental conditions

Before working on the decision task, participants were randomly assigned to one of three experimental conditions or to a control condition. Participants in the control condition completed the task as described above without any decision aids (NoAid). In two of the experimental conditions participants were provided with standardized information sheets (SIS*)*, either instead (SISonly) or in addition to (SISadd) having the possibility to engage with the web platform as described above. The SIS was modeled after official SIS formats and contained key information deemed essential for informed decision-making (see FigureS1). The SIS presented data in standardized table format, including details about costs, service coverage, and contract duration periods. To facilitate comparison, each provider's complete contract portfolio was displayed in a single table using consistent formatting. For more focused analysis, participants could also access individual SIS versions that displayed information for a single contract while maintaining the same standardized format. In the third experimental condition, a total cost calculator was added (TCCadd*)* alongside the information provided via the web platform. Participants could easily toggle between the main website and the TCC using a button in the top right corner of the screen. The TCC was built on our defined parameters for essential contract features and optimal decision-making, ensuring its calculations aligned with these criteria (detailed in the decision quality section). To use the TCC, participants entered their minimum requirements for call minutes, text messages, and data usage. Upon clicking 'calculate,' the system generated a cost-ordered list of eligible contracts, with the most cost-effective option appearing first (see Figure 1). The results displayed monthly costs and included allowances for each service (minutes, texts, and data). Participants could access additional contract details—such as overage charges, activation fees, service charges, and data speeds—by selecting a 'details' button for any listed option.

## Measures

## Decision quality

To measure decision quality, we calculated the average monthly cost for each offer, taking into account a range of costs such as the monthly fee, the activation fee, coverage costs, service fees or discounts over a two-year period based on a specific usage pattern (user profile). The offers were structured in a way that one offer stood out as the optimal solution, having the lowest monthly cost for the given user profile. Due to the positively skewed distribution of absolute incremental costs, we converted the absolute values into ranks from 1 to 20, with 20 representing the lowest incremental cost (i.e., the optimal decision) and values closer to 1 suggesting lower quality decisions.

## Consumer confusion

We measured the degree to which participants experienced information overload with eight items taken from the consumer confusion scale (Kasper et al., 2010). Ratings were made on a 6-point scale (1 = *applies not at all* and 6 = *applies completely)* and averaged into a single score (*α* = .90) with higher values representing greater confusion (e.g., “The more I learn about cellular service contracts and providers, the more difficult becomes my choice.”).

## Systematic motivation

We included systematic motivation as an exploratory variable. Following previous research (e.g., de Dreu, 2003; Scholten et al., 2007), we measured systematic motivation with four items (e.g. “I tried to take into consideration all possible alternatives.”). Answers were given on a 6-point-scale (1 = *applies not at all*, 6 = *applies completely*) and averaged into a single measure (α = .79) with higher scores representing higher systematic motivation.

# Results

## Decision Quality

To examine the effect of experimental conditions on decision quality, we conducted a Kruskal-Wallis test. The initial analysis revealed no significant effect of experimental condition, H(3) = 3.9, *p* = .269. However, we identified 38 participants (18%) that had not utilized the decision aids provided. After excluding these participants, the effect of experimental condition was significant, H(3) = 9.2, *p* = .027, ƞ² = .04. Pairwise comparisons with Mann-Whitney tests revealed that individuals in the TCCadd condition (*M* = 17.35, *SD* = 4.04, *MR)* = 53.4) made better decisions compared to individuals in the SISonly condition (*M* = 14.98, *SD* = 5.33, *MR* = 38.9; *Z* = -2.66, *p* = .008, *r* = -.19). Similarly, individuals in the SISadd condition made better decisions (*M* = 16.63, *SD* = 5.37, *MR* = 50.6) than individuals in the SISonly condition (*M* = 14.98, *SD* = 5.33, *MR* = 38, *Z* = -2.32, *p* = .021, *r* = -.16). TCCadd and SISadd did not differ from the control condition, although decision quality was slightly lower in the latter (*M* = 15.90, *SD* = 5.19, *MR* = 84.1).

## Consumer Confusion

We hypothesized that experimental condition would influence consumer confusion. However, a one-way ANOVA revealed no significant differences in consumer confusion across conditions. Additionally, we found no meaningful relationship between consumer confusion and decision quality, *r* = -.05, *p* = .483.

## Systematic motivation

We ran a one-way ANOVA to test whether the use of a certain decision aid had an effect on systematic motivation. The analysis revealed that systematic motivation differs between experimental conditions, *F*(3, 197) = 4.26, *p* = .006, η^2^= .061. Post-hoc test revealed a significant difference in systematic motivation between SISonly (M = 4.79, SD= 1.02) and TCCadd (M = 4.05, SD = 1.09, p = .003).

# Discussion

This prestudy provides initial insights into the effectiveness of different decision aiding techniques on consumer decision quality, consumer confusion, and systematic motivation in a complex multi-dimensional choice task. The results offer guidance for designing a more comprehensive main study while suggesting preliminary implications for consumer protection regulation.

We hypothesized that decision aids (standardized-information-sheets, total cost calculator) enhance decision quality and reduce consumer confusion. While the initial analysis showed no significant differences between conditions, the emergence of significant effects after excluding non-users of decision aids highlights the crucial role of actual tool engagement. Both TCCadd and SISadd conditions demonstrated superior decision quality compared to the SISonly condition, suggesting that adding decision aids to the conventional decision environment may indeed improve consumer outcomes when properly utilized. While decision aid designs affected decision quality, they did not significantly impact consumer confusion levels. Furthermore, the absence of a correlation between consumer confusion and decision quality challenges assumptions about their relationship. This suggests that reducing confusion alone may not be sufficient to improve decision outcomes. Interestingly, adding a CDA seems to reduce systematic motivation. The reduced systematic motivation in the calculator-based decision aid condition, while seemingly counterintuitive, aligns with the fundamental purpose of CDA: to require fewer cognitive resources by presenting key information in an organized, readily accessible format. By automating complex calculations and highlighting critical data points, these aids allow decision-makers to reach quality decisions while

One of the most intriguing findings of this prestudy is that a considerable proportion of participants was reluctant to use the decision aids. The emergence of significant effects only after excluding these non-users raises important questions about decision aid acceptance and effectiveness. The non-usage could stem from various factors, such as insufficient salience of the decision aids, lack of perceived usefulness, or possible technical barriers or usability issues. It also raises the question of whether this reluctance is predictable. As many individuals were able to make good decisions even without decision aids in the control condition, it is important to explore the role of individual differences in information processing preferences that might explain this differential.

## Conclusion

Initial evidence suggests that adding decision aids to the conventional decision environment can improve decision quality. The significant proportion of non-users in this prestudy highlights a critical challenge in decision aid implementation. While the aids showed promise when used, their effectiveness is meaningless if consumers don't engage with them. These initial results suggest that policy development should focus not just on decision aid design, but on the entire ecosystem of factors that influence their acceptance and effectiveness. Understanding and addressing the barriers to decision aid usage will be crucial for realizing their potential benefits for consumer decision-making.

References

Breugelmans, E., Koehler, C. F., Dellaert, B. G. C., and deRuyter, K. (2012). Promoting interactive decision aids on retail websites: A message framing perspective with new vs. traditional focal actions. *Journal of Retailing* 88, 226–235. doi: 10.1016/j.jretai.2011.10.003.

Bröder, A., and Schiffer, S. (2006). Stimulus format and working memory in fast and frugal strategy selection. *Journal of Behavioral Decision Making* 19, 361–380. doi: 10.1002/bdm.533.

de Dreu, C. K. (2003). Time pressure and closing of the mind in negotiation. *Organizational Behavior and Human Decision Processes* 91, 280–295. doi: 10.1016/S0749-5978(03)00022-0.

Diehl, K., Kornish, L. J., and Lynch, J. G. (2003). Smart agents: When lower search costs for quality information increase price sensitivity. *Journal of Consumer Research* 30, 56–71. doi: 10.1086/374698.

Elie‐Dit‐Cosaque, C. (2015). “Information Overload,” in *Wiley Encyclopedia of Management*, ed. C. L. Cooper (Wiley), 1–4.

Faul, F., Erdfelder, E., Lang, A.-G., and Buchner, A. (2007). G*Power 3: A flexible statistical power analysis program for the social, behavioral, and biomedical sciences. *Behavior Research Methods* 39, 175–191.

Garcia, C., and van Boom, W. H. (2009). Information Disclosure in the EU Consumer Credit Directive: Opportunities and Limitations. *SSRN Journal.* doi: 10.2139/ssrn.1538111.

Häubl, G., and Trifts, V. (2000). Consumer Decision Making in Online Shopping Environments: The Effects of Interactive Decision Aids. *Marketing Science* 19, 4–21. doi: 10.1287/mksc.19.1.4.15178.

Helberger, N. (2013). Form matters: Informing consumers effectively. *SSRN Journal.* doi: 10.2139/ssrn.2354988.

Jacoby, J., Speller, D. E., and Kohn, C. A. (1974). Brand choice behavior as a function of information load. *Journal of Marketing Research* 11, 63–69. doi: 10.2307/3150994.

Kasper, H., Bloemer, J., and Driessen, P. H. (2010). Coping with confusion: The case of the Dutch mobile phone market. *Managing Service Quality* 20, 140–160. doi: 10.1108/09604521011027570.

Kasper, J., and Lenz, M. (2005). Criteria for the development and evaluation of decision aids. *German Journal for Quality in Health Care* 99, 359–365.

Lee, B.-K., and Lee, W.-N. (2004). The effect of information overload on consumer choice quality in an on-line environment. *Psychology and Marketing* 21, 159–183. doi: 10.1002/mar.20000.

Lee, J., and Cho, J. (2005). Consumers’ use of information intermediaries and the impact on their information search behavior in the financial market. *Journal of Consumer Affairs* 39, 95–120. doi: 10.1111/j.1745-6606.2005.00005.x.

Lee, M., Carswell, C. M., Seidelman, W., and Sublette, M. (2012). The design of product comparison tables and its effects on decision making. *Proceedings of the Human Factors and Ergonomics Society Annual Meeting* 56, 1654–1658. doi: 10.1177/1071181312561331.

Murray, K. B., and Häubl, G. (2007). “Interactive consumer decision aids,” in *Handbook of marketing decision models*, ed. B. Wierengar (New York: Springer US), 55–77.

Oehler, A. (2012). Klar, einfach, verständlich und vergleichbar: Chancen für eine standardisierte Produktinformation für alle Finanzdienstleistungen - Eine empirische Analyse. *Zeitschrift für Bankrecht und Bankwirtschaft* 24, 119–133. doi: 10.15375/zbb-2012-0204.

Oehler, A., and Reisch, L. A. (2008). Behavioral Economics – eine neue Grundlage für Verbraucherpolitik? Eine Studie im Auftrag des Verbraucherzentrale Bundesverbandes.

Scholten, L., van Knippenberg, D., Nijstad, B. A., and Dreu, C. K. de (2007). Motivated information processing and group decision-making: Effects of process accountability on information processing and decision quality. *Journal of Experimental Social Psychology* 43, 539–552. doi: 10.1016/j.jesp.2006.05.010.

Stone, D. N., and Schkade, D. A. (1994). Effects of attribute scales on process and performance in multiattribute choice. *Organizational Behavior and Human Decision Processes* 59, 261–287. doi: 10.1006/obhd.1994.1060.

Vogrincic-Haselbacher, C., Krueger, J. I., Lurger, B., Dinslaken, I., Anslinger, J., Caks, F., Florack, A., Brohmer, H., and Athenstaedt, U. (2021). Not Too Much and Not Too Little: Information Processing for a Good Purchase Decision. *Frontiers in psychology* 12, 642641. doi: 10.3389/fpsyg.2021.642641.

# Supplementary figures

**Supplementary Figure 1.** Example view of the Standardized information sheet (SIS).
