# Supplementary figures and images for: Enhancing decision quality through computer-based decision aids: how promotional interventions and Need for Cognition shape effectiveness in online consumer choices
Source: Front Psychol. 2025 Oct 29;16:1576319. doi: 10.3389/fpsyg.2025.1576319 (PMC12605097; doi:10.3389/fpsyg.2025.1576319)

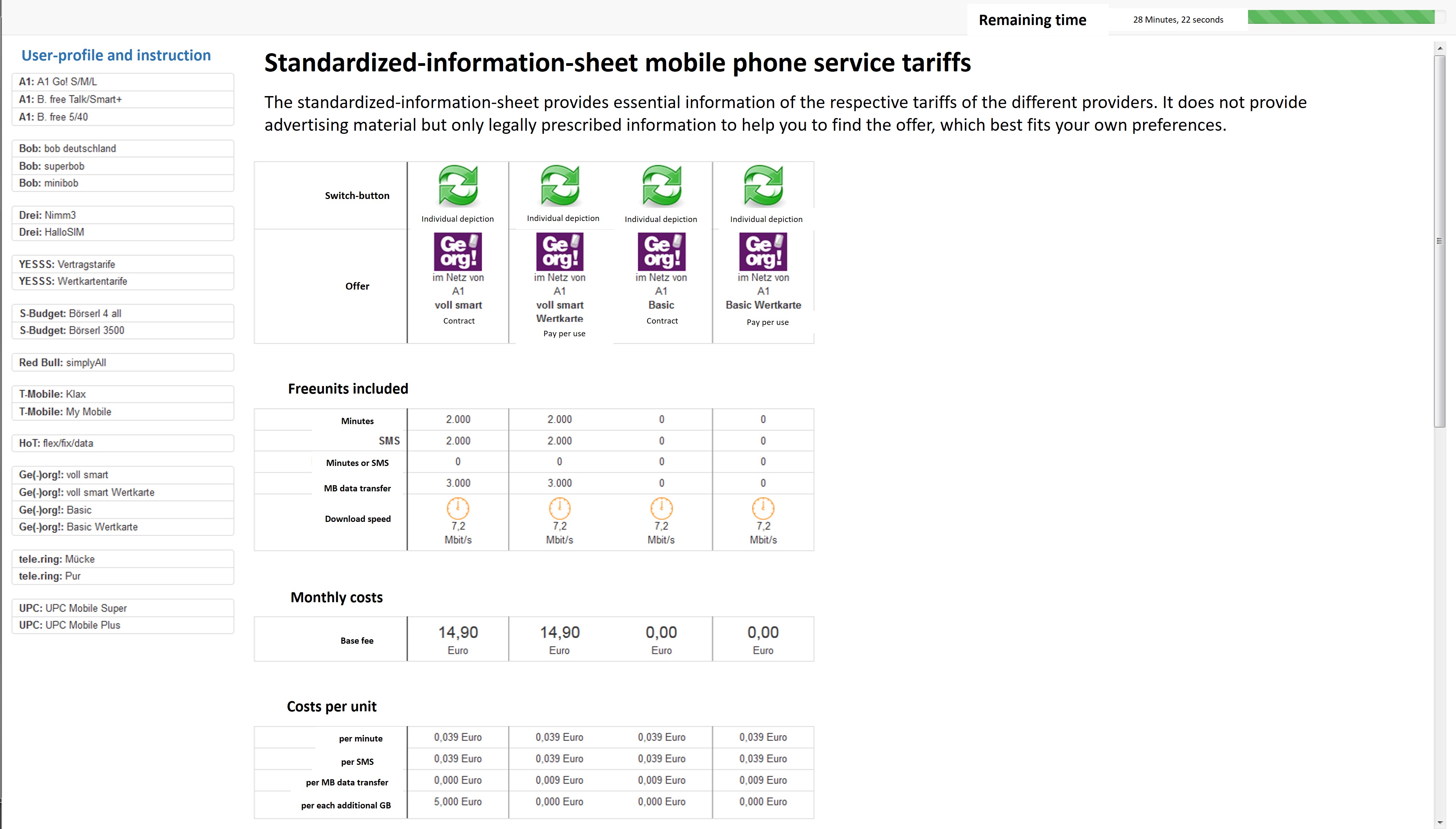

Supplement: Supplementary file 2 [file Image_1.jpeg]
